# Transcriptomic Analysis Identifies A Tolerogenic Dendritic Cell Signature

**DOI:** 10.3389/fimmu.2021.733231

**Published:** 2021-10-20

**Authors:** Harry Robertson, Jennifer Li, Hani Jieun Kim, Jake W. Rhodes, Andrew N. Harman, Ellis Patrick, Natasha M. Rogers

**Affiliations:** ^1^ Kidney Injury Group, Centre for Transplant and Renal Research, Westmead Institute for Medical Research, Westmead, NSW, Australia; ^2^ Computational Systems Biology Group, Children’s Medical Research Institute, Westmead, NSW, Australia; ^3^ School of Mathematics and Statistics, University of Sydney, Camperdown, NSW, Australia; ^4^ Centre for Virus Research, Westmead Institute for Medical Research, Westmead, NSW, Australia; ^5^ The University of Sydney, School of Medical Sciences, Faculty of Medicine and Health Sydney, Sydney, NSW, Australia; ^6^ Renal and Transplantation Medicine, Westmead Hospital, Westmead, NSW, Australia; ^7^ Thomas E. Starzl Transplantation Institute, Department of Surgery, University of Pittsburgh School of Medicine, Pittsburgh, PA, United States

**Keywords:** dendritic cell, tolerogenic dendritic cell (tolDC), gene expression profile analysis, mature dendritic cells, mononuclear phagocyte cells, transcriptomic, liver, human dendritic cell

## Abstract

Dendritic cells (DC) are central to regulating innate and adaptive immune responses. Strategies that modify DC function provide new therapeutic opportunities in autoimmune diseases and transplantation. Current pharmacological approaches can alter DC phenotype to induce tolerogenic DC (tolDC), a maturation-resistant DC subset capable of directing a regulatory immune response that are being explored in current clinical trials. The classical phenotypic characterization of tolDC is limited to cell-surface marker expression and anti-inflammatory cytokine production, although these are not specific. TolDC may be better defined using gene signatures, but there is no consensus definition regarding genotypic markers. We address this shortcoming by analyzing available transcriptomic data to yield an independent set of differentially expressed genes that characterize human tolDC. We validate this transcriptomic signature and also explore gene differences according to the method of tolDC generation. As well as establishing a novel characterization of tolDC, we interrogated its translational utility *in vivo*, demonstrating this geneset was enriched in the liver, a known tolerogenic organ. Our gene signature will potentially provide greater understanding regarding transcriptional regulators of tolerance and allow researchers to standardize identification of tolDC used for cellular therapy in clinical trials.

## Introduction

Dendritic cells (DC) represent a population of bone marrow (BM)-derived cells responsible for the collection and presentation of captured antigen (Ag) (1). DC are found throughout the body, and their capacity for Ag presentation provides a crucial link between innate and adaptive immune responses. Multiple DC subsets have been described, broadly divided into myeloid and plasmacytoid groups (2). Similar to other immune cells, DC are also able to alter their phenotype and function based on environmental cues (3), contextual inflammatory signaling, and the presence of self/non-self Ag. Classically, mature DC drive effector T cell responses, and immature DC mediate central or peripheral tolerance primarily through immunoregulatory factors that induce regulatory or anergic T cells (4). An additional subset that are maturation-resistant – so-called tolerogenic DC (tolDC) – can be manufactured *ex vivo* but have not yet been found physiologically. TolDC have been extensively interrogated in pre-clinical models, and are exceedingly effective at limiting host immune responses that drive autoimmune disease [summarized in (5)] or allograft rejection in transplantation [summarized in (6)]. Capitalizing on their ability to modulate T and/or B cell behavior and release immunomodulatory molecules, tolDC have been used in recent phase I/II clinical trials for type 1 diabetes (7), rheumatoid arthritis (8), multiple sclerosis (9), and liver and kidney transplantation (10) as therapeutic agents that reduce exposure to non-specific immunosuppressive drugs.

Multiple protocols for the generation of tolDC exist (11). BM-derived progenitors (animals) and CD14+ peripheral blood mononuclear cells (PBMC, humans) are driven towards prototypic DC using growth factor/cytokine cocktails, and then “tolerized” pharmacologically. Interleukin-10 (IL-10) and vitamin D-based regimens are most frequently used, a substantial list of pharmacological modifiers of DC function exists (12) which continues to expand (6). Avoiding *ex vivo* isolation and manipulation, *in vivo* modulation using DC-specific targeting techniques, such as nanoparticles (13, 14) or antibodies (15), can directly deliver a pharmacological payload. Despite treatment heterogeneity, the DC phenotype is characterized by immunoregulatory properties (16) which then assumes generation of stable tolDC.

Identification of DC subsets is typically based on cell-surface markers. Although expression appears relatively conserved between species, tissues and disease models (2), the same standardized characteristics are not yet available for tolDC. Indeed, tolDC used in recent clinical studies did not have uniform methods for generation, phenotype or functional measurements (17). To date, there is no consensus for “gold-standard” validation of tolerogenic properties, and current methods range from analysis of cell-surface markers to allogeneic T cell stimulation (10). This has significant implications for clinical trials where differences in tolDC generation may impact clinical outcomes. There is also ongoing concern that tolDC are not stably manipulated and, like regulatory T cells, can be subverted to activated or inflammatory forms by a permissive microenvironment. Understanding gene changes that robustly reflect tolDC would be a useful tool in standardizing their generation, which may ultimately impact patient outcome.

Transcriptomic analysis allows for the identification of conserved and differentially expressed genes in tolDC regardless of the method of generation. A specific transcriptomic signature may also assist with discovery of surrogate markers that may be used clinically. The adaptation of differentially expressed genes to enrichment pathways also provides insight into the biological interpretability of gene(s) of interest. Recent literature (18) seeking to bridge this gap in the literature are limited to consolidating already reported signatures of previous studies and drawing on published conclusions to extract a transcriptome unique to the tolDC phenotype. We have addressed this shortcoming by analyzing available datasets to yield an independent set of differentially expressed genes within each study. Comparing these results across datasets yielded a common tolDC transcriptome which we then validated. We used the same pipeline to generate a mature DC transcriptome, and both novel gene signatures were applied to immune cell populations *in vivo*.

## Methods

### TolDC Data Acquisition

**Figure 1:**
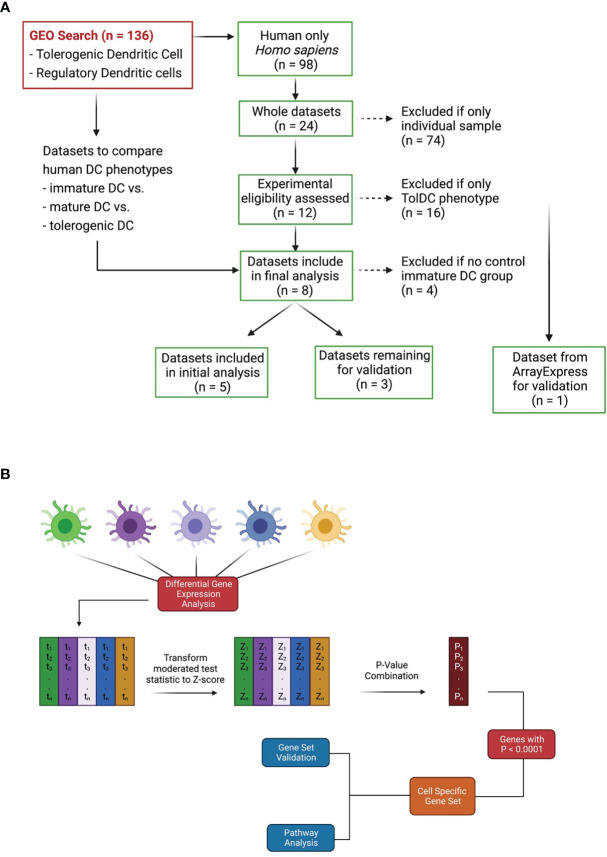
Dataset identification and workflow for tolDC gene analysis. **(A)** Flowchart demonstrating relevant GEO search with inclusion and exclusion criteria. **(B)** Pipeline for generating tolDC, AADC and mature DC gene signatures.

A search to identify publicly available gene expression data in the Gene Expression Omnibus (GEO) https://www.ncbi.nlm.nih.gov/geo/ was performed using the terms: “tolerogenic dendritic cell”, “regulatory dendritic cell” and “tolDC”. The search for publications up to December 2020 revealed 136 Datasets, of which 98 were human. Datasets were initially excluded from downstream analysis if they did not have an immature DC phenotype (control) within the dataset. Only 24 were whole datasets, and 8 contained cell samples that included adequately phenotyped tolDC (**Figure 1A**). These datasets were arbitrarily divided into two groups: 5 datasets were used for initial tolDC gene set discovery, and the 3 remaining were used for validation. One further validation dataset was obtained from ArrayExpress (19).

### Data Analysis

The raw data of each of the five datasets precured [GSE13762 (20), GSE23371 (21), GSE56017 (22), GSE117946 (23), GSE52894 (24)] were obtained from the gene expression omnibus (https://www.ncbi.nlm.nih.gov/geo/). All five datasets were normalized using the quantile normalization method, with each dataset filtered to exclude genes with nil expression. Within each dataset, differential gene expression analysis was performed using limma (Smyth G. K. 2004) with Benjamini–Hochberg multiple testing correction (*p* < 0.05). In this way, a moderated test statistic was calculated for each gene within each dataset. Moderated test statistics were converted to z-scores, and subsequently p-values, as described in the directPA vignette (25). Pearson’s method of combining p-values was used to derive an overall significance score for each gene across all datasets (**Figure 1B**). An overall significance score of p < 0.00001 was used as the threshold to establish genes in the tolDC transcriptome.

### TolDC Gene Signature Validation

Three (3) datasets acquired from GEO (GSE104438 (26), GSE98480 (27), GSE92852 (28) containing tolDC and immature DC gene expression data were used for validation. A final validation was also performed using data from ArrayExpress database (E-MTAB-6937 (19). As with our discovery and initial validation set, we analysed each dataset individually to diminish potential batch effects that would arise from merging datasets. In all datasets, the moderated test statistics for each gene were converted into z-scores (as outlined in **Figure 1B**) and the pattern of gene expression compared with our tolDC gene set.

### Alternatively Activated Dendritic Cell Gene Signature

In a similar manner to the identification of genes critical to tolDC, we determined genes differentially expressed between the tolDC stimulated with and without lipopolysaccharide (LPS). Three datasets were used in the analysis: GSE23371 (21), GSE117946 (23), GSE52894 (24). Differential gene expression was performed using the limma pipeline optimized as above, combining the results of our analyses using Pearson’s Method, and yielding a set of genes critical to defining AADC.

### Mature DC Gene Signature

Differentially expressed genes between immature DC stimulated with and without lipopolysaccharide (LPS) were also explored. Four datasets were used in the analysis: GSE23371 (21), GSE56017 (22), GSE117946 (23), GSE52894 (24).

### Analysis of Enriched Pathways

A Wilcoxon rank sum test was performed on the combined p-value that was determined for each gene within our gene set analysis, returning a significance value for KEGG pathways that were enriched in the DC of interest. A subsequent Gene Set Enrichment Analysis (GSEA) was performed on the ranked list of genes, executed using the clusterProfliler (29) package in R.

### Signature Validation

We sought to validate the specificity of our mature and tolDC signature using *in vivo* datasets that contained mononuclear phagocytes (MNP), including recognized DC subsets (30, 31) or peripheral blood immune cell subsets [GSE28492 (32)]. RNAseq data was normalized using the TMM method without filtering, microarray data was normalized using quantile normalization, and gene expression was compared between each cell phenotype.

### Single Cell RNAseq of Kidney, Liver, and PBMC Datasets

Five individual single cell RNAseq (scRNA-seq) samples were obtained from the Panglao database (https://panglaodb.se/). The search criteria were initially limited to liver tissue only from human donors. The accession code SRA716608 was used to extract scRNAseq into R for analysis. The five samples were normalized and integrated using the harmony algorithm. The combined dataset was then analysed using the Uniform Manifold Approximation and Projection (UMAP) dimensional reduction technique. The tolDC phenotype was then plotted on the UMAP projection. To compare tolDC and mature DC gene signatures in different tissue compartments, liver (SRA716608, n = 22154 cells), peripheral blood mononuclear cells (PBMC, SRA749327, n = 15881 cells) and kidney cortex (SRA598936, n = 3573 cells) scRNA-seq samples were also acquired. Datasets belonging to individual tissue types were integrated using the harmony method, normalized and scaled. The expression of genesets was measured between DC in each tissue type.

### Data Availability and Code Statement

Data utilized for this study is publicly available using the GEO accession codes listed. The code utilized to generate analysis and figures is available at: https://github.com/Harry25R/Transcriptomic-analysis-identifies-a-tolerogenic-dendritic-cell-signature.git.

## Results

### Dataset Quality Control

**Figure 2:**
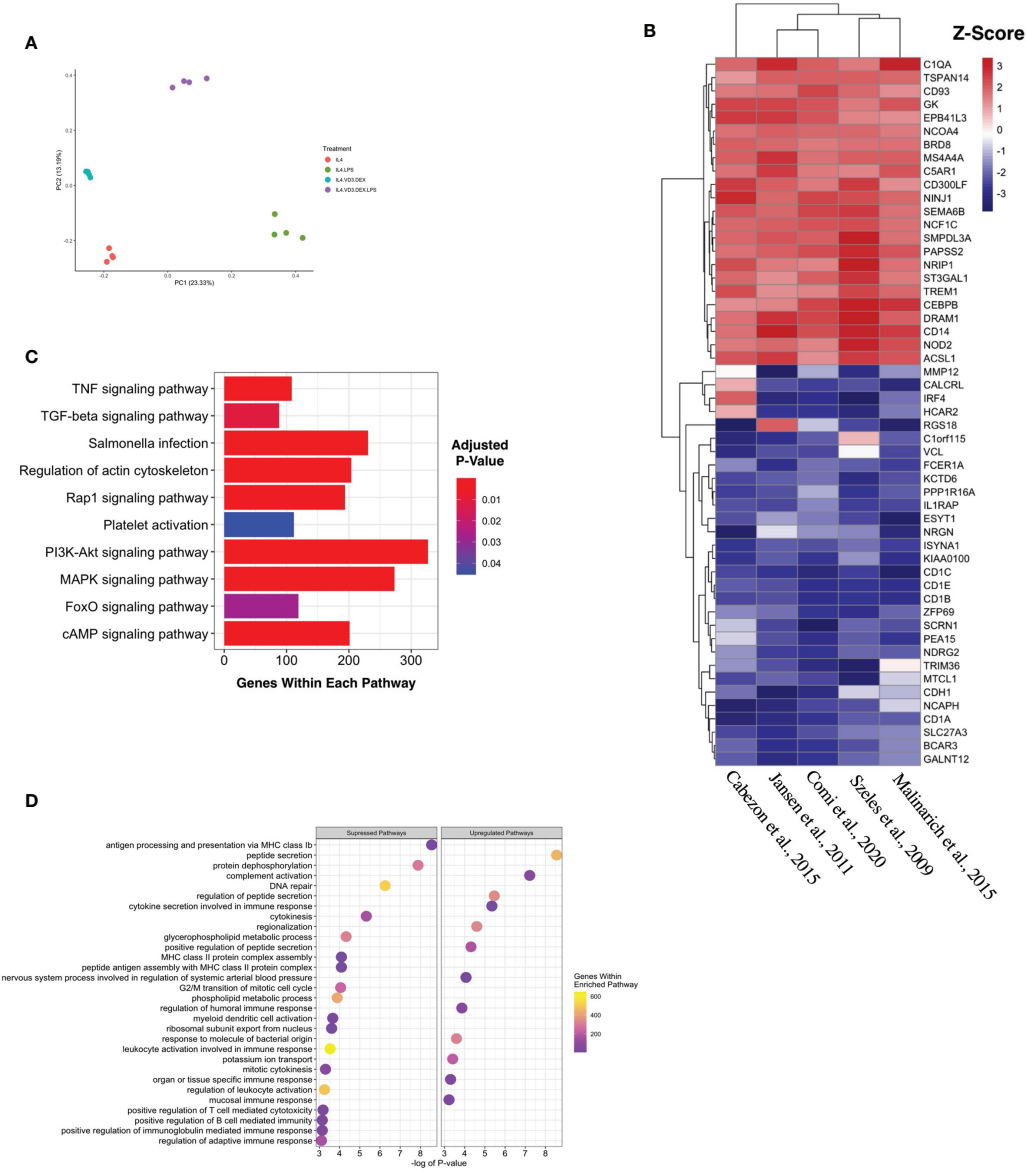
Generating a unique tolDC transcriptome. **(A)** Principal component analysis (PCA) plot characterizing change in gene expression profiles between immature DC (red), mature DC (green), tolDC (blue), or alternatively-activated tolerogenic DC (AADC, purple) in GSE52894. Each dot presents a sample, and each color represents a DC phenotype. **(B)** Heatmap representation of the top 20 differentially expressed genes (DEG) by tolDC. DEG were arranged by hierarchical clustering on the vertical axis. Datasets, also clustered by hierarchical clustering, are displayed on the horizontal axis. The p-value yielded from each study were converted to z-scores and plotted. **(C)** KEGG and **(D)** Gene Set Enrichment analyses. Each point on the dot plot represents the number of genes involved in the relevant pathway. The gene ratio is the proportion of DEG *versus* genes not differentially expressed. Each point was colored to represent the adjusted p-value using the Benjamini-Hochberg method.

Five complete datasets with tolDC gene sequencing were retrieved. Each dataset had a different method of tolDC generation and 3 studies shared the same sequencing platform (**Table 1**). A principal component analysis (PCA) identified phenotypic specific differences between samples in the GSE52894 dataset (24) (**Figure 2A**). This was consistent across all included datasets (**Supplementary Figure 1A**). Across the first principal component we observed large differences when DC were matured with LPS. The largest source of variation was between tolerogenic and mature DC, an expected result given the regulatory nature of tolDC compared to mature (immunogenic) DC. Confirming these results, unsupervised hierarchical clustering between samples exhibited strong correlation between samples of the same phenotype (**Supplementary Figure 1B**).

**Table 1:** Identified publicly available gene datasets including immature, tolerogenic and mature DC for initial tolDC gene set discovery.

Dataset ID	Platform ID	References	Sample Proportions	Agent Used to Induce the tolDC Phenotype
GSE13762	GPL570	(20)	4 x imDC, 8 x tolDC	Vitamin D
GSE23371	GPL570	(21)	3 x imDC 3 x imDC + LPS 3 x tolDC 3 x tolDC + LPS	Interleukin 10 & Dexamethasone
GSE56017	GPL570	(22)	6 x imDC 6 x imDC + LPS 6 x imDC + Dexamethasone 6 x tolDC	Dexamethasone
GSE117946	GPL6244	(23)	4 x imDC 4 x imDC + LPS 4 x tolDC 4 x tolDC + LPS	Interleukin 10
GSE52894	GPL10558	(24)	4 x imDC 4 x imDC + LPS 4 x tolDC 4 x tolDC + LPS	Dexamethasone & Vitamin D

### Establishing a tolDC Gene Signature

The results of individual differential gene expression analysis were ranked by p-value. The top 10 up-regulated and downregulated genes are listed in **Tables 2A**, **2B**, respectively. Our results were consistent with previous reports, suggesting no homogeneity in differentially expressed genes DEG between different methods generating tolDC if only looking at the strongest changes (18). By considering more than just the top genes, we then assessed homogeneous differential gene expression across the datasets, identifying 53 genes with a combined p-value<10^-5^ which we deemed to be characteristic of tolDC (**Table 3**). The top 20 DEG are displayed in heatmap form (**Figure 2B**).

**Table 2A:** Top 10 differentially upregulated genes in tolDC.

Dataset (GEO ID)	Method of Generation	Number of DE Genes	Top 10 DE Gene (Upregulated)
**GSE13762**	Vitamin D	77	SHE, CYP24A1, DRAM1, ST6GAL1, CD2AP, NRIP1, AOAH, G0S2, C20orf197, MIR3945HG
**GSE23371**	Interleukin 10 & Dexamethasone	140	RNASE1, S100A8, CD163, SELENOP, CD14, SLC18B1, LINC01094, MERTK, C1QB, ADAMDEC1
**GSE56017**	Dexamethasone	218	TNFAIP6, CCL20, C17orf58, NFKBIA, KYNU, PNRC1, SOD2, TNFAIP3, CYTIP, STK26
**GSE117946**	Interleukin 10	68	FAM20A, IGF2BP3, FPR1, HIVEP2, CR1, FCGR3A, C1S, CD163, IL7, TGFA
**GSE52894**	Dexamethasone & Vitamin D	196	C20orf197, UBASH3B, SLC37A2, CA2, COQ2, FBP1, SIGLEC6, LRRC8A, ST6GAL1, ATP5PF

**Table 2B:** Top 10 differentially downregulated genes in tolDC.

Dataset (GEO ID)	Method of Generation	Number of DE Genes	Top 10 DE Gene (Downregulated)
**GSE13762**	Vitamin D	77	IRF4, IER3, TRIM36, SPIN4, HCAR2, MMP12, CH25H, WFDC21P, CD1e, NUCB2
**GSE23371**	Interleukin 10 & Dexamethasone	140	MMP12, ALOX15, CDH1, CH25H, APOL4, LAMP3, CCL17, MAFF, ACOT7, SOCS1
**GSE56017**	Dexamethasone	218	RGS18, TSPAN32, NRGN, NCAPH, KIAA0930, C11orf45, CD1a, ACOX2, LPCAT4, DDIAS
**GSE117946**	Interleukin 10	68	SCRN1, B3GNT5, PLPP1, CD1c, HCAR3, TIFAB, ATP1B1, MAP4K1, CDH1, FABP4
**GSE52894**	Dexamethasone & Vitamin D	196	SLC47A1, CD1c, ESYT1, RGS18, ABCA6, DHRS2, CLIP2, HLA-DMB, DOCK10, CALCRL

**Table 3:** Summary of differentially expressed genes in tolDC.

Upregulated Genes	Downregulated Genes
DRAM1, NRIP1, CEBPB, SMPDL3A, NOD2, CD14, PAPSS2, ST3GAL1, SEMA6B, CD300LF, ACSL1, TREM1, NINJ1, NCF1C, RGS18, TSPAN14, MS4A4A, CD93, NCOA4, BRD8, C1QA, GK, C5AR1, EPB41L3	IRF4, TRIM36, MTCL1, HCAR2, MMP12, KCTD6, ZFP69, PP1R16A, CD1A, CD1E, CD1B, CD1C, IL1RAP, ESYT1, CALCRL, NCAPH, BCAR3, PEA15, FCER1A, SCRN1, GALNT12, NDRG2, ISYNA1, SLC27A3, NRGN, KIAA0100, VCL, CDH1, C1orf115

### TolDC Pathway Enrichment Analysis

Mapping DEG within the tolDC gene set to the KEGG database returned several enriched pathways (**Figure 2C**). The mitogen-activated protein kinase (MAPK) pathway was significantly enriched, as were cyclic AMP, Ras-related Protein 1, Forkhead box O and tumor necrosis factor (TNF) pathways. Gene Set Enrichment Analysis (GSEA) assigned directional change to each pathway and ranked genes were then mapped against the Gene Ontology (GO) database. Encouragingly, pathways involved in antigen presentation and antigen binding were all suppressed (**Figure 2D**), consistent with literature demonstrating that tolDC negatively regulate the immune response.

### TolDC Gene Set Validation

**Figure 3:**
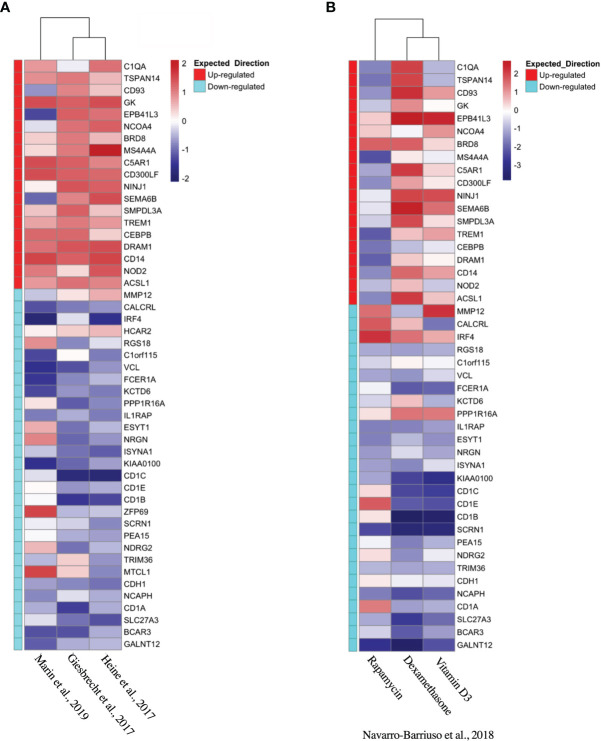
Validation of tolDC transcriptome. Heatmap representation of upregulated and downregulated genes from the tolDC discovery gene set compared to expression in **(A)** GEO-derived or **(B)** ArrayDatabase validation gene set.

Based on the initial discovery set, we identified 3 appropriate gene sets for validation (**Table 4**), annotating each gene by the expected enrichment direction (**Figure 3A**). Our gene signature fit data from TLR- and interluekin-10-generated tolDC, although GM-CSF-generated tolDC performed poorly in this validation step. We conducted further validation of our tolDC gene set using data from (19) (**Table 4**) who compared transcriptomic signatures from tolDC derived from 3 different treatments (vitamin D, dexamethasone or rapamycin). Rapamycin-derived tolDC demonstrated a significant genomic deviation from our gene signature (**Figure 3B**).

**Table 4:** Identified publicly available gene datasets including immature, tolerogenic and mature DC for tolDC gene set validation.

Dataset ID	Platform ID	References	Sample Proportions	Agent Used to Induce the tolDC Phenotype
GSE104438	GPL14550	(26)	4 x Macrophage, 4 x imDC 4 x tolDC	Low dose GM-CSF
GSE98480	GPL10558	(27)	3 x imDC 3 x imDC + LPS 3 x tolDC 3 x imDC + Poly I:C	Toll like receptor 7/8 ligand (R848)
GSE92852	GPL18460	(28)	3 x imDC 3 x imDC + LPS 3x tolDC 3 x tolDC + LPS	Interleukin-10
E-MTAB-6937 (ArrayDatabase)	–	(19)	5 x imDC 5 x imDC + LPS 5 x rapa-tolDC 5 x dexa-tolDC 5 x vitD3-tolDC	Rapamycin Dexamethasone Vitamin D

### Alternatively-Activated tolDC

**Figure 4:**
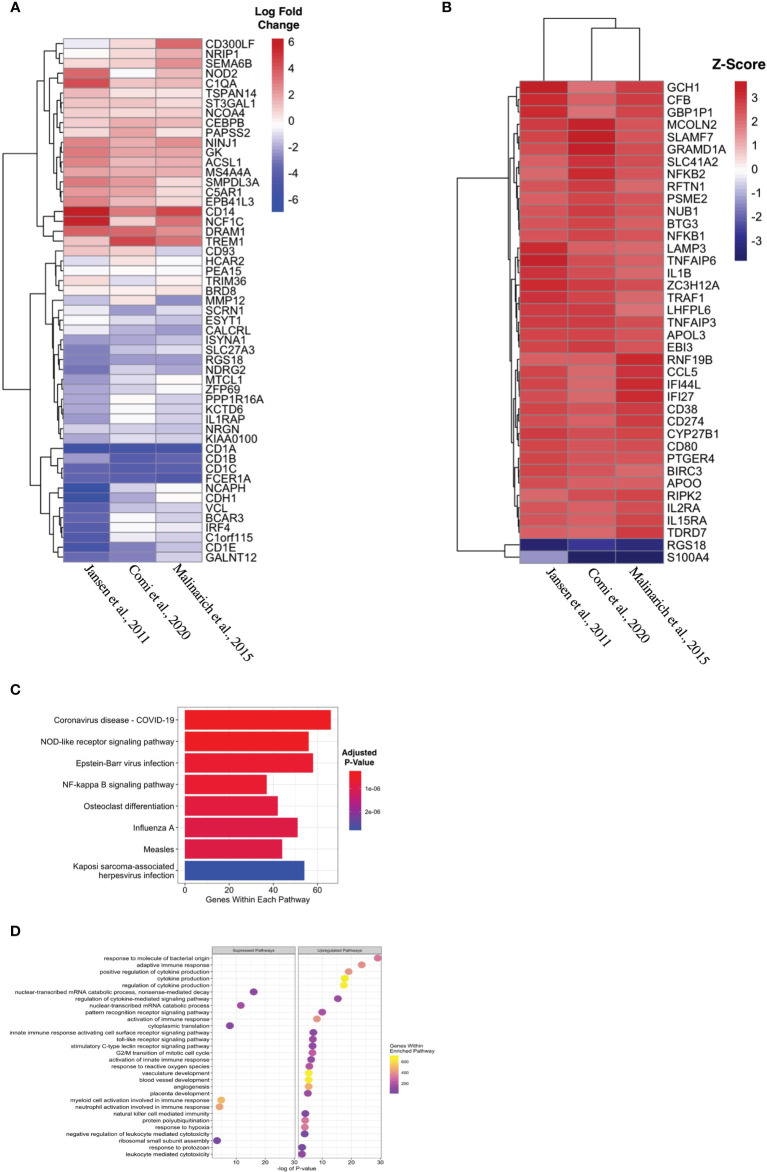
Identifying transcriptomic differences between subtypes of tolDC. **(A)** Heatmap representation of the top 39 DEG by AADC. **(B)** Fold change difference in expression of genes in AADC compared to tolDC. **(C)** KEGG and **(D)** Gene Set Enrichment analyses.

Propagated tolDC that are “alternatively activated” (AADC) by exposure to an inflammatory stimulus, typically LPS, also demonstrate robust regulatory properties that protect against graft-*versus*-host disease (33, 34). AADC have shown greater efficacy in controlling inflammatory immune responses *in vivo (* 35) compared to a more modest effect from IL-10-conditioned tolDC (36). We initially interrogated three datasets that compared gene expression between AADC and tolDC, although these demonstrated different DEG (**Figure 4A**, **Table 5**). Analysis determined 39 DEG that were enriched in AADC compared to tolDC (**Table 4** and **Figure 4B**), and we mapped these to GEA pathways (**Figures 4C, D**).

**Table 5:** Summary of differentially expressed genes in AADC.

Upregulated Genes	Downregulated Genes
BTG3, NF-KB1, NF-KB2, RFTN1, SLC41A2, SLAMF7, GRAMD1A, LHFPL6, NDP, MCOLN2, PSME2, IFI27, IFI44L, RNF19B, GCH1, GBP1P1, APOO, CCL5, CD274, CYB27B1, G0S2, CD38, CD80, CFB, TNFAIP6, ZC3H12A, TNFAIP3, APOL3, NUB1, LAMP3, IL-1B, TRAF1, EBI3, PTGER4, BIRC3, RIPK2, IL2RA, IL15RA, TDRD7	RGS18, S100A4

### DC Signatures in Tissue

**Figure 5:**
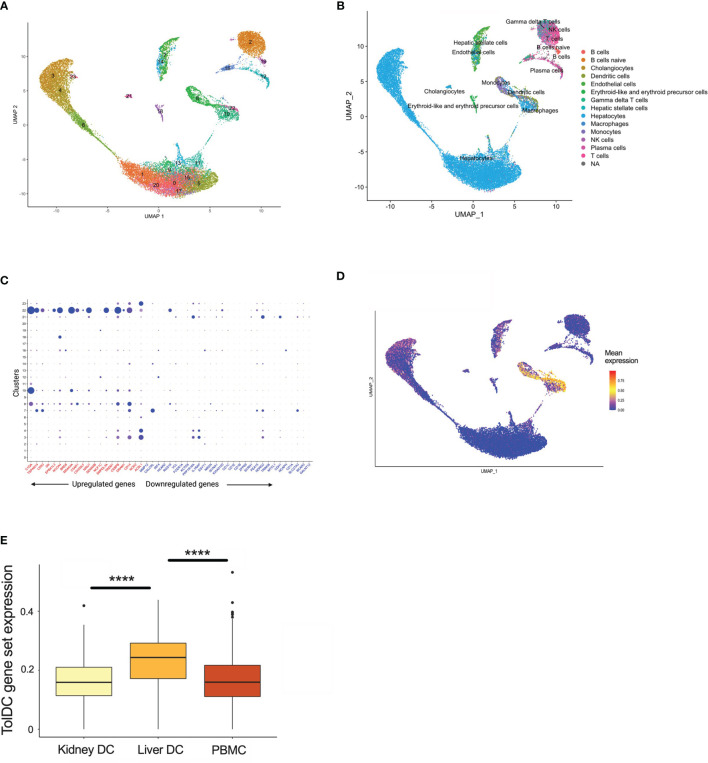
TolDC gene set is overexpressed in liver-resident DC. **(A)** UMAP plot displaying the clustering of harmony integrated scRNAseq samples. **(B)** UMAP plot of liver datasets annotated by cell type. **(C)** Dot plot displaying up- and down-regulated tolDC gene expression markers enriched within cell clusters. **(D)** UMAP plot demonstrating a joint density analysis of upregulated genes from the tolDC gene set. **(E)** Boxplot displaying the expression of the tolDC gene set across tissue-resident and circulating DC. ****p < 0.0001.

The liver is unique amongst solid organs in its capacity to modulate local and systemic tolerance. This is contributed to by the presence of unconventional antigen presenting cells (liver sinusoidal endothelial cells, Kupffer cells) (37), altered T cell proportions (particular γδ subsets) (38, 39), and an increased ratio of DC to parenchymal cells (2-5 times higher in liver compared to other organs) (40). Importantly, liver-resident DC demonstrate features most consistent with a tolerogenic phenotype and function, with low endocytic capacity, decreased MHC expression, limited T cell allostimulation and high IL-10 production (41–43). Using scRNAseq samples from healthy human liver which has been clustered by cell type (**Figure 5A**, **Supplementary Figure 2A**), we then demonstrated that upregulated genes within the tolDC signature was enriched in areas which mapped to DC/monocyte/macrophage lineage within the liver (**Figures 5B–D**). Downregulated genes were not overexpressed in any cell type (**Supplementary Figure 2B**). We also interrogated whether our tolDC signature was overexpressed in the kidney (which has significantly lower tolerogenic capacity) and/or PBMC. We were able to demonstrate that our gene signature was not enriched in either compared to liver (**Figure 5E**), although an analysis of housekeeping genes (44) was not significantly different (**Supplementary Figure 2C**).

### The Relevance of DC Gene Signatures *In Vivo*

**Figure 6:**
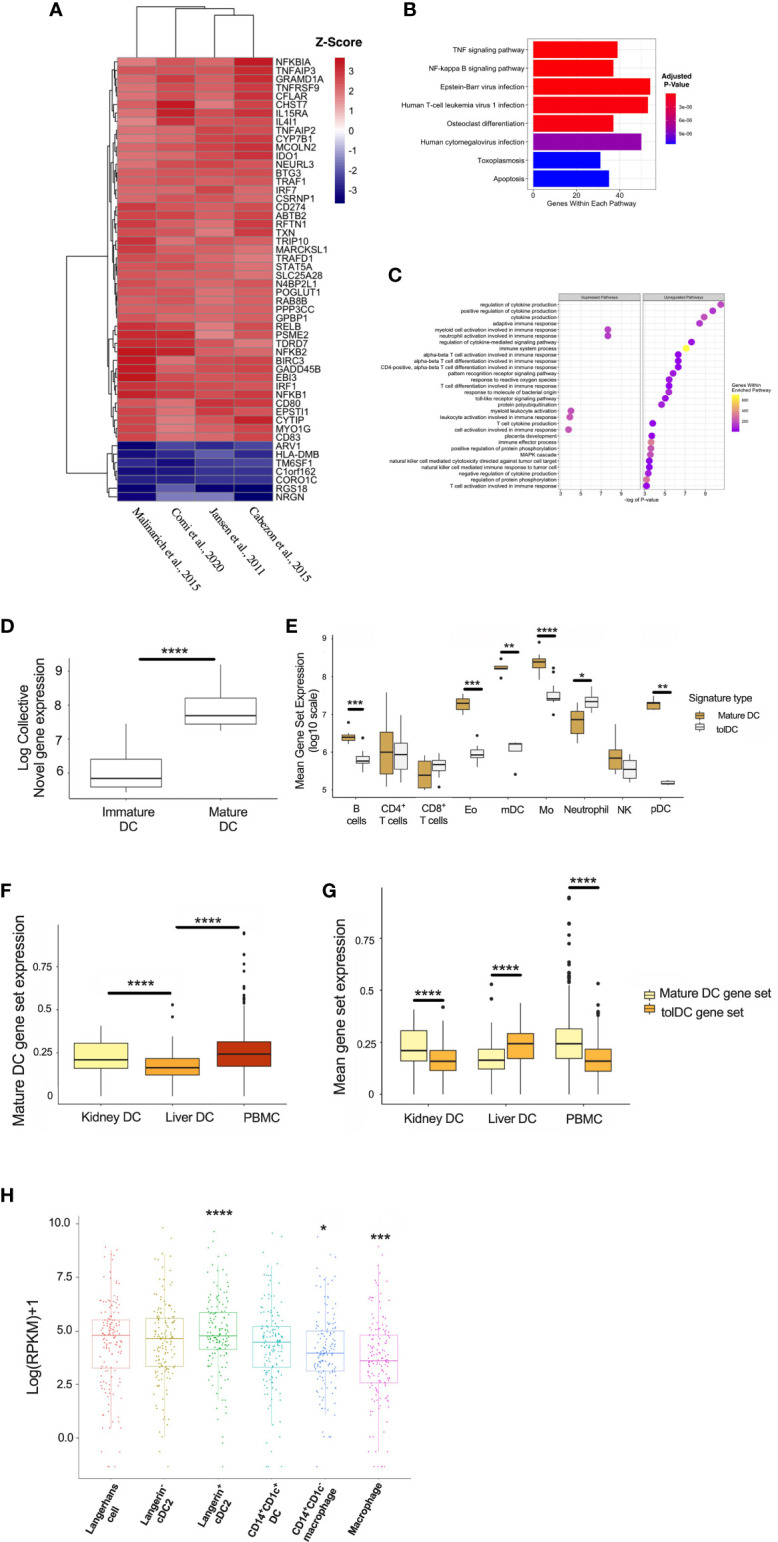
Generating a mature DC transcriptome. **(A)** Heatmap representation of the top 52 DEG within the mature DC phenotype. **(B)** KEGG and **(C)** Gene Set Enrichment Analyses. **(D)** Mature DC gene-set expression in myeloid cell subsets isolated from epithelial tissues. **(E)** Comparison of tolDC and mature DC gene signatures in peripheral blood immune cell subsets. **(F)** Boxplot displaying the expression of genes critical to mature DC across DC in liver, kidney and PBMC. **(G)** Comparison of tolDC and mature DC gene set expression in liver, kidney and PBMC. **(H)** Mononuclear phagocytes from epithelial and subepithelial tissues were isolated and classified as DC or macrophage. The average expression of the mature DC gene signature was plotted between cells. A two-sample t-test was performed to determine statistically significant differences in base mean expression of the mature DC gene set across MNP. *p < 0.05, **p < 0.01, ***p < 0.001, ****p < 0.0001.

DC are rare populations within the peripheral blood (45), but reside at greater frequency within tissue interstitial compartments in an immature state, and sample the environment in organs exposed to potential (neo-)antigens in lung (46, 47), kidney (48), and skin (49, 50). The potential for exogenous stimuli to initiate DC activation suggests that the mature DC gene signature might be enriched in tissue-specific DC subsets *in vivo*. A total of 64 genes were significantly differentially expressed between the mature and immature DC, and the top 52 genes were heat-mapped (**Figure 6A**). The enrichment analysis yielded pathways relevant to cell inflammation and infection (**Figures 6B, C**). Mature DC are well-defined in the literature, and the correlation with an inflammatory gene signature demonstrates the reliability of our pipeline to resolve genes according to DC phenotype, as well as supporting the current hypothesis that DC are influenced by the surrounding environment (3).

To further demonstrate the physiological relevance of our DC gene signatures, we used a dataset identifying 6 myeloid cell subsets (31), demonstrating that our mature DC gene set correlated with the appropriate (mature) DC subset identified *in vivo* (**Figure 6D**). This also shows our approach to identifying a cell-specific gene signature on microarray platforms could be successfully applied to RNAseq data. Interestingly, the tolerogenic and mature DC gene sets could also be applied to distinct immune cell subsets within peripheral blood (32), with the latter enriched in myeloid DC (mDC) and monocytes (**Figure 6E**). We applied our mature DC signature to liver, kidney and PBMC scRNAseq samples, demonstrating significantly lower expression in liver (**Figure 6F**). Kidney-resident DC and PBMC showed an enhanced mature DC signal compared to tolDC (**Figure 6G**). We also interrogated a recent dataset comparing the expression profiles of mononuclear phagocytes (MNP) isolated from epidermal and dermal tissue (30). The expression of our mature, but not tolerogenic, DC signature was significantly higher in recognized DC subsets (**Figure 6H** and **Supplementary Figures 3A, B**).

## Discussion

Here we derive novel, distinct genetic signatures for both tolDC and mature DC. Both gene sets align with known biological differences in phenotype and function, and can be used to identify physiological DC subsets *in vivo*. Most interesting was the mapping of the tolDC signature to liver DC. Our analysis also demonstrated that tolDC and immature DC are distinct subsets, despite current paradigms suggesting overlap of several features (51), and these data support the notion that tolDC indeed derive from specific transcriptional programming.

We identified several genes critical to tolDC function. Several compartments of the CD1 glycoprotein complex were downregulated in the tolDC gene set. CD1 is a cell surface protein that is involved in presentation of lipid-based antigens to T-cells and natural killer cells that subsequently mediate adaptive immunity (52, 53). CD1 autoreactive T-cells, particularly CD1a and CD1c, are abundant among circulating T-cells from healthy human adults and neonates (54) and are associated with a variety of diseases. The plasticity of CD1 antigen presentation highlights evolved mechanisms that regulate the self/non-self cellular lipid environment presented to T‐cells. With CD1a-c expression decreased in the tolDC we can speculate defective T cell stimulation ability due to altered antigen processing and presentation (55). This finding has also been replicated in tissue-resident CD103+ conventional DC which were less effective in antigen cross-presentation with accumulated lipid bodies (56).

CD14, a known monocyte cell-surface marker in blood, is expressed by tissue-based macrophages, and was significantly upregulated in tolDC. CD14 has several functions on the surface of monocytes, ranging from metabolism to pathogen-associated-molecular pattern (PAMP) identification in the innate immune response (57). CD14 binds to extracellular LPS and acts as a secondary receptor to TLR4 in facilitating a subsequent immune response (58). However, recent data has demonstrated that DC subsets expressing CD14 impeded T-cell proliferation (59). Interestingly, CD14 and CD1a kinetics are replicated in human monocyte-derived DC whose maturation capacity are limited by co-culture with immune complexes (60).

The global gene expression profile of tolDC identified prominent enrichment of the mitogen-associated protein kinase (MAPK) pathway. This finding is in keeping with reports that MAPK (specifically p38) inhibition promotes an immunogenic DC phenotype (61) and augments effector T cell responses (62). Cytoskeletal pathway changes (specifically related to actin filaments) were suppressed, a process that is fundamental to plasma membrane internalization for endocytosis and vesicle transportation required for antigen processing and cell surface presentation (63, 64).

Our tolDC gene set was validated in datasets from publications generating tolerogenic human DC using a variety of pharmacological agents (TLR ligands, IL-10, vitamin D and dexamethasone). TolDC propagated using GM-CSF (26) or rapamycin (19) demonstrated noticeably different transcriptomes, in keeping with known phenotypic and functional differences (although direct *in vitro* comparisons were not consistently reported). GM-CSF alone is not commonly used *in vitro* for this purpose, and has been shown to produce tolDC that are distinct from the established literature, including greater plasticity (65) and metabolic changes that drive T cell inhibition (26). Rapamycin-induced tolDC also diverge from other tolDC, producing higher bioactive IL-12 and lower IL-10 levels (66), in addition to strikingly discrepant findings of mTOR inhibition on DC function that demonstrate activation (67, 68) or inhibition (69, 70).

Alternatively-activated DC (AADC), tolerogenic DC activated by inflammatory stimuli, are effective in inducing anergic and regulatory T cell responses (34) that protects against lethal graft-*versus*-host-disease in pre-clinical models (33). Only 3 comparative datasets were available for analysis and did not demonstrate homogeneity between DEG from AADC and tolDC. Gene enrichment analysis demonstrated increased virus and stress responsiveness, as well as cytokine-signaling/inflammatory pathways, with concurrent downregulation of mitochondrial function. Metabolic plasticity, including enhanced catabolism, has been correlated with DC function, and our findings correlated with previous work demonstrating decreased oxidative phosphorylation capacity with LPS-stimulated tolDC (24).

TolDC are artificially generated *in vitro*, and therefore not wholly representative of DC found physiologically. However, natural and induced DC with tolerogenic capacity (71) are crucial for homeostatic function, particularly in tissues exposed to environmental stimuli. The liver is considered the most tolerogenic organ, and our tolDC gene signature was overrepresented in four integrated scRNAseq datasets of healthy human liver, clustering with liver-resident DC (with overlap seen in the macrophage/monocyte population). DC and macrophages are interrelated, derive from common lineages, and are often phenotypically and functionally indistinguishable (51). Hepatic DC are distinct from other tissue-based DC (37, 72), abundantly secreting immunosuppressive cytokines (41, 73) that dictate immunoregulatory properties. We mapped the tolDC gene set to scRNAseq samples of healthy (and more immunogenic) kidney as a comparator, but the signature was not overexpressed, in keeping with clinical and experimental data that support organ-specific differences in allograft acceptance (74).

Our pipeline generating a tolDC transcriptomic signature was applied to developing a gene set relevant to mature DC. Genes deemed significant to mature DC were strongly implicated in the inflammatory response and, using the KEGG database, mapped to TNF-α and NF-kB signaling pathways. NF-κB is a central mediator of pro-inflammatory gene induction and functions in both innate and adaptive immune cells, and central for DC maturation (75). We were able to demonstrate that our mature DC gene signature was enriched in CD1c+ mature DC rather than CLEC9A+ immature DC. These findings, while not novel, speak to the validity of our methods in characterizing DC phenotype using gene expression datasets, and demonstrate that our signature could be applied to physiological DC *in vivo*.

This paper further highlights the need for further -omic studies to identify a consensus gene expression profile, including distinct signaling pathways, that can confirm tolDC function and stability *in vivo*. Despite the reported safety of tolDC in early-phase human trials (17), and known efficacy in large animal models (76), potential variability in clinical grade tolDC preparations remains a concern for translational purposes. The advent of standardized tolDC manufacturing through Focus and Accelerate Cell-based Tolerance-inducing Therapies (77) aims to minimize variations in approach and is a key step towards a standardized tolDC production for pre-clinical studies and clinical trials. Understanding the genomic processes behind the functional properties of DC and identification of molecular targets of immunomodulation provide potential opportunities for intervention to silence unwanted immune responses.

## Data Availability Statement

Publicly available datasets were analysed in this study. This data can be found here: GSE13762, GSE23371, GSE56017, GSE117946, GSE52894, GSE104438, GSE98480, GSE92852, ArrayExpress database E-MTAB-6937.

## Author Contributions

NR and EP led the study, designed experiments, and wrote the manuscript. HR, JL, and EP analysed the data. All authors contributed to experimental design and drafting the manuscript.

## Funding

JL is supported by a NHMRC postgraduate scholarship (GNT1168776). AH is supported by National Health Medical Research Council (NHMRC) Ideas Grant (GNT1181482). EP is supported by a Discovery Early Career Researcher Award from the Australian Research Council. NR is supported by NHMRC Project and Career Development Grants (GNT1138372, GNT1158977 respectively).

## Conflict of Interest

The authors declare that the research was conducted in the absence of any commercial or financial relationships that could be construed as a potential conflict of interest.

## Publisher’s Note

All claims expressed in this article are solely those of the authors and do not necessarily represent those of their affiliated organizations, or those of the publisher, the editors and the reviewers. Any product that may be evaluated in this article, or claim that may be made by its manufacturer, is not guaranteed or endorsed by the publisher.

## Supplementary Material

Supplementary Figure 1A Principal component analysis (PCA) from tolDC discovery and validation datasets. PCA plots characterizing the change in gene expression profiles between immature DC (red), mature DC (green), tolDC (blue), or alternatively-activated tolerogenic DC (AADC, purple).

Supplementary Figure 1B Heatmap demonstrating the correlation between samples. Gene signatures from immature DC (blue), mature DC (red), tolDC (purple), and AADC (green) from relevant datasets. Both the horizontal and vertical axis were clustered using the same hierarchical clustering algorithm. Each square on the heatmap is the value of Pearson’s correlation coefficient between the two sample, and values are assigned a color.

Supplementary Figure 2 TolDC gene signature expression in human kidney tissue. **(A)** UMAP plot of human liver datasets integrated using the harmony method. Each dataset was annotated using the accessible code on the Panglao database. **(B)** UMAP plot demonstrating a joint density analysis of downregulated genes from the tolDC gene set. **(C)** Boxplot displaying the expression of housekeeping genes glyceraldehyde 3-phosphate dehydrogenase (GAPDH), succinate dehydrogenase complex subunit A (SDHA) and peptidylprolyl isomerase A (PPIA) across liver and kidney DC, and PBMC.

Supplementaary Figure 3 TolDC gene set expression in human mononuclear phagocytes. Mononuclear phagocytes were isolated from epithelial and sub-epithelial tissues. The average expression of the tolDC gene signature was plotted between cells. The average expression of the tolDC gene signature was plotted between cells. A two-sample t-test was performed to determine differences in base mean expression of the tolDC gene set across MNP. **(B)** Boxplot displaying differences between tolDC and mature DC transcriptomic signatures within each MNP subset. **p < 0.01, ***p < 0.001.
