# Supplementary figures and images for: Transcriptomic Analysis Identifies A Tolerogenic Dendritic Cell Signature
Source: Front Immunol. 2021 Oct 20;12:733231. doi: 10.3389/fimmu.2021.733231 (PMC8564488; doi:10.3389/fimmu.2021.733231)

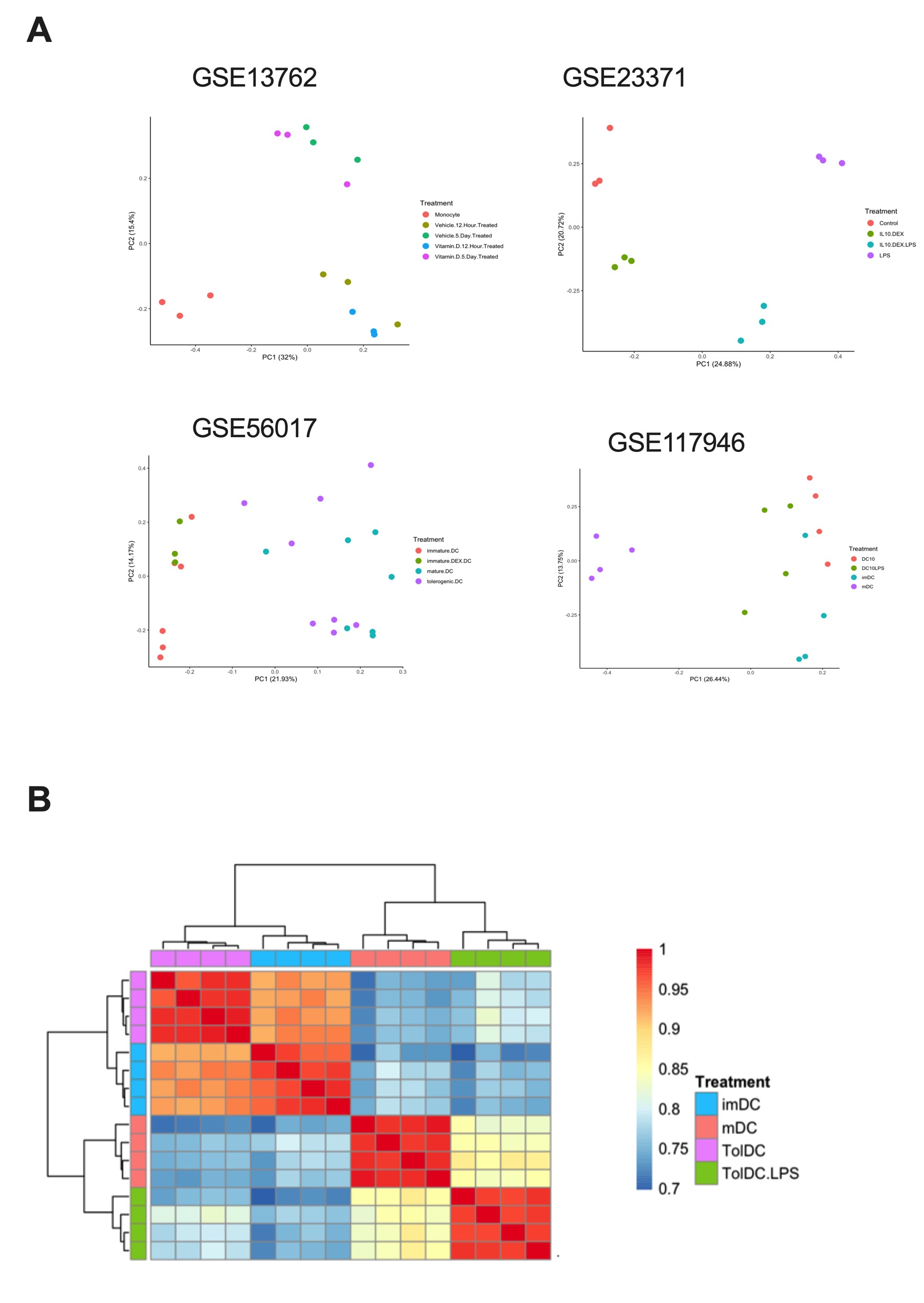

Supplement: Supplementary Figure 1A — Principal component analysis (PCA) from tolDC discovery and validation datasets. PCA plots characterizing the change in gene expression profiles between immature DC (red), mature DC (green), tolDC (blue), or alternatively-activated tolerogenic DC (AADC, purple). [file Image_1.jpeg]

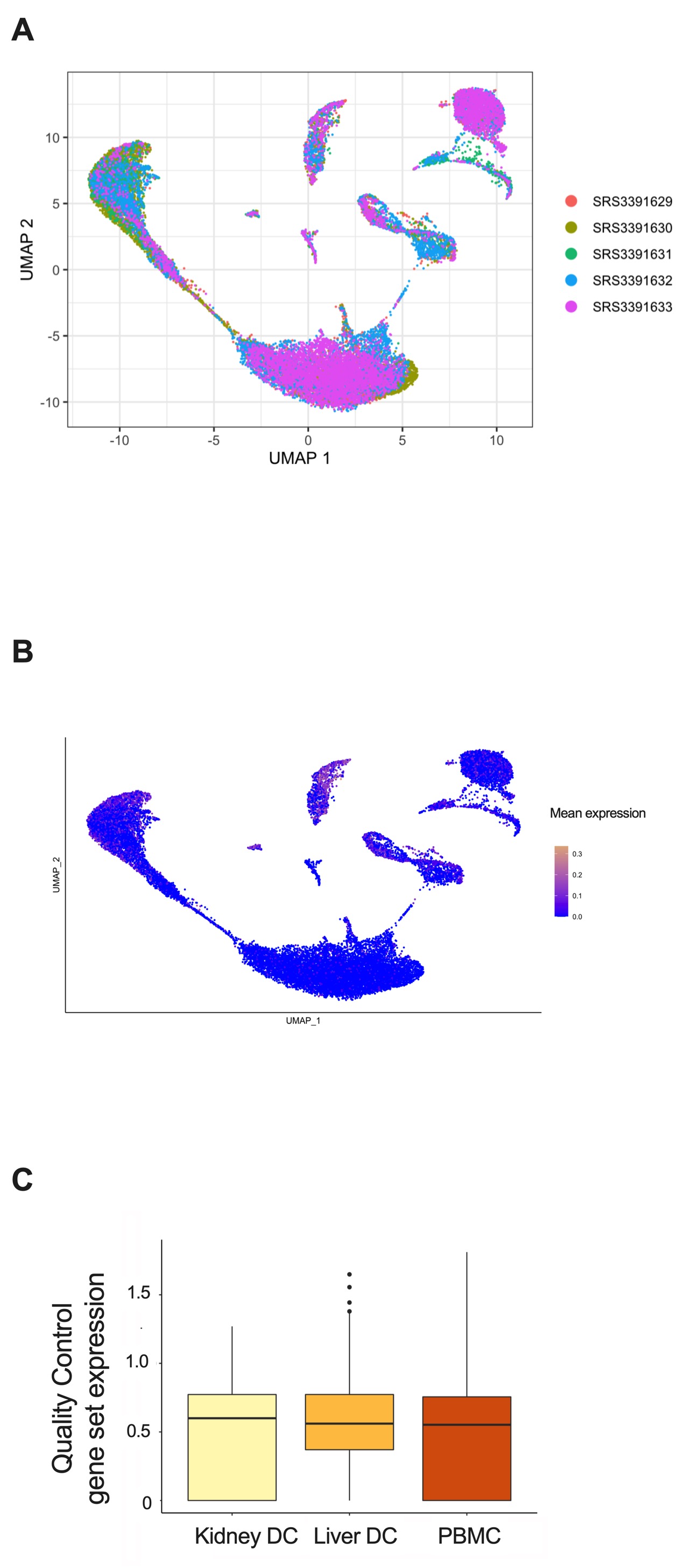

Supplement: Supplementary Figure 2 — TolDC gene signature expression in human kidney tissue. (A) UMAP plot of human liver datasets integrated using the harmony method. Each dataset was annotated using the accessible code on the Panglao database. (B) UMAP plot demonstrating a joint density analysis of downregulated genes from the tolDC gene set. (C) Boxplot displaying the expression of housekeeping genes glyceraldehyde 3-phosphate dehydrogenase (GAPDH), succinate dehydrogenase complex subunit A (SDHA) and peptidylprolyl isomerase A (PPIA) across liver and kidney DC, and PBMC. [file Image_2.jpeg]

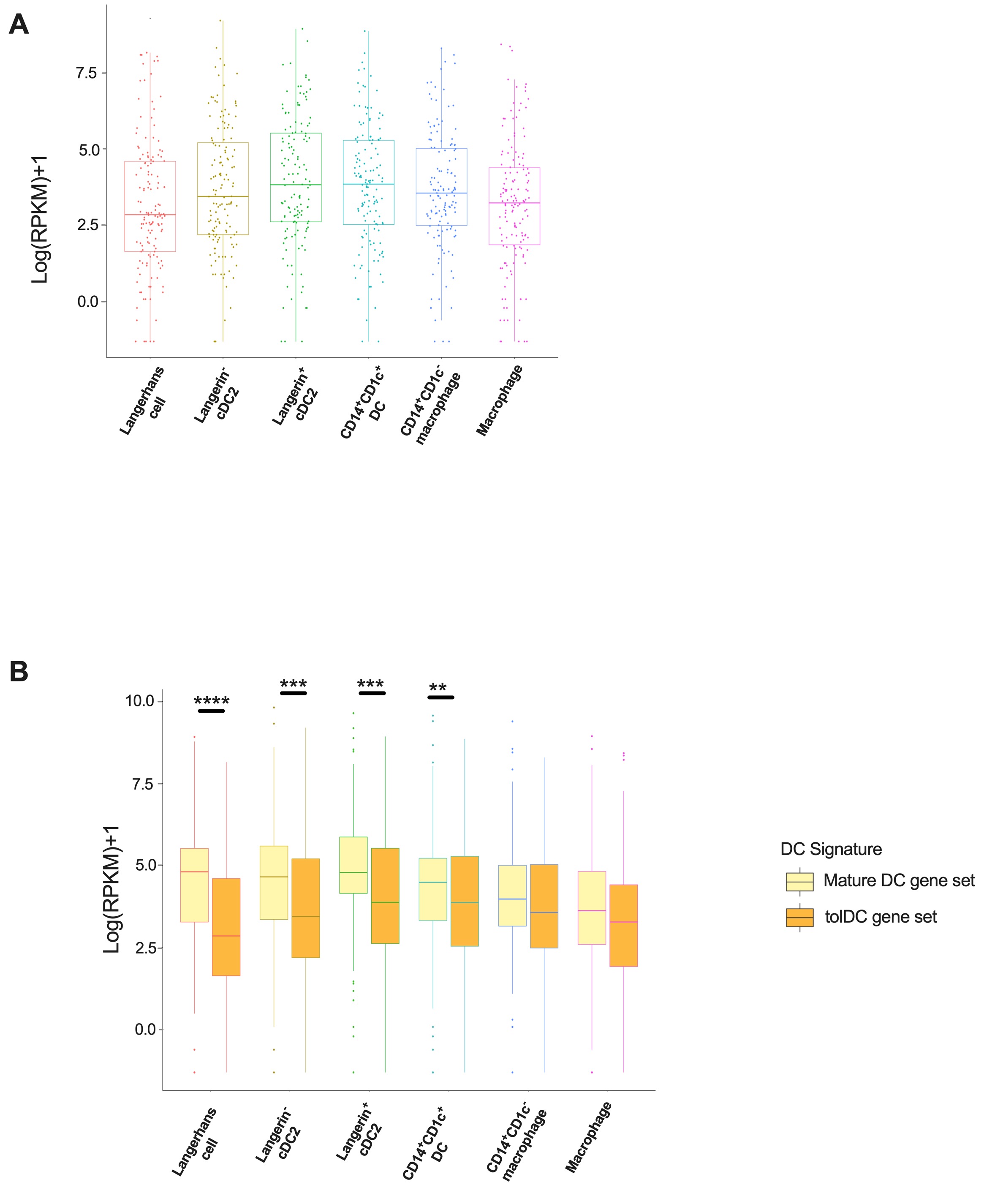

Supplement: Supplementaary Figure 3 — TolDC gene set expression in human mononuclear phagocytes. Mononuclear phagocytes were isolated from epithelial and sub-epithelial tissues. The average expression of the tolDC gene signature was plotted between cells. The average expression of the tolDC gene signature was plotted between cells. A two-sample t-test was performed to determine differences in base mean expression of the tolDC gene set across MNP. (B) Boxplot displaying differences between tolDC and mature DC transcriptomic signatures within each MNP subset. **p < 0.01, ***p < 0.001. [file Image_3.jpeg]
